# Selected Biopolymers’ Processing and Their Applications: A Review

**DOI:** 10.3390/polym15030641

**Published:** 2023-01-26

**Authors:** María Flórez, Patricia Cazón, Manuel Vázquez

**Affiliations:** Department of Analytical Chemistry, Faculty of Veterinary, University of Santiago de Compostela, 27002 Lugo, Spain

**Keywords:** biopolymer, casting, coating, electrospinning, 3D printing, compression molding, graft copolymerization

## Abstract

Petroleum-based polymers are used in a multitude of products in the commercial world, but their high degree of contamination and non-biodegradability make them unattractive. The development and use of polymers derived from nature offer a solution to achieve an environmentally friendly and green alternative and reduce waste derived from plastics. This review focuses on showing an overview of the most widespread production methods for the main biopolymers. The parameters affecting the development of the technique, the most suitable biopolymers, and the main applications are included. The most studied biopolymers are those derived from polysaccharides and proteins. These biopolymers are subjected to production methods that improve their properties and modify their chemical structure. Process factors such as temperature, humidity, solvents used, or processing time must be considered. Among the most studied production techniques are solvent casting, coating, electrospinning, 3D printing, compression molding, and graft copolymerization. After undergoing these production techniques, biopolymers are applied in many fields such as biomedicine, pharmaceuticals, food packaging, scaffold engineering, and others.

## 1. Introduction

Humans have utilized biopolymers made from agricultural materials including wool, cotton, leather, and silk for a very long time. However, waste derived from synthetic polymer is growing alarmingly. It was in the 1920s that these agricultural resources began to be used in the industrial sector for fuels and consumer products. After World War II, these biopolymers were replaced by chemically derived products because they are more cost effective, strong, and versatile [1]. According to data provided by the European Commission, almost 26 million tons of plastic-derived wastes are generated only in Europe every year. In addition, around 80% of marine litter is derived from these plastics (European Commission, https://environment.ec.europa.eu/topics/plastics_en, (accessed on 14 December 2022)). Depletion of petroleum resources, expanding environmental regulations, shortage of landfill space, and emissions from incineration are the main problems arising from the use of plastics. Furthermore, as petroleum resources are finite, their costs are likely to rise. Economic and environmental concerns are working together to accelerate the production of new environmentally beneficial materials [1,2,3]. 

In recent decades, interest in the development of the so-called “biopolymers” has increased. The main advantages of these materials are that they reduce the production of carbon dioxide, and their biodegradability reduces the amount of waste sent to landfills [4]. Biopolymers are biological macromolecules formed by the polymerization of monomers such as: proteins (polymers of amino acids), nucleic acid (polymers of nucleotide), and polysaccharides (polymers of sugars) [5]. Synthetic polymers are made up of similar and random structures, whereas biopolymers have a well-defined structure [6]. Natural polymers often have low C-C bond energies, making them vulnerable to degradation. Unlike most synthetic polymers, natural fibers degrade under the influence of the environment, including air, light, heat, and microorganisms [7]. They are renewable resources with exceptional qualities including low density and weight, cost effectiveness, and ease of production. Moreover, natural polymers are now preferred over synthetic because they process more slowly, cause less damage to machinery and its components, and do not contaminate land or water [8]. The replacement of non-biodegradable synthetic materials with biodegradable materials is of such importance in our society that it is estimated that the production of biopolymers will reach 2.87 million tons in 2024 (European Bioplastics, 2020, https://www.european-bioplastics.org/market/ (accessed on 14 December 2022)). 

The properties of natural-based materials vary depending on the processing method and treatment applied to the biopolymer, the material matrix and composition, and environmental parameters [9]. For instance, the moisture affinity and tendency to agglomerate are the main problems regarding the use of polysaccharide-based materials [10]. The manufacturing processes used to create components from conventional plastics, such as electrospinning, graft copolymerization, 3D impression, etc., are typically the same for natural polymers. In this review, the processing techniques for the production of natural polymers are exposed and discussed. Moreover, the optimal conditions for developing the production method and the most commonly used biopolymers and their applications are presented.

## 2. Bio-Based Polymers Directly Extracted from Biomass

Bio-based materials are made from renewable resources including wood, agricultural waste, natural fiber plants, grasses, etc., which are rich in proteins, polysaccharides, lipids, and other compounds [3]. Biopolymers are mainly classified according to the source from which they are derived.

One of the main sources of biopolymers are polysaccharides, which are the most abundant renewable resource in the world, since these molecules make up most of the terrestrial biomass, exerting structural functions in plants (cellulose or pectin) and in animal skeletons (chitin). Some molecules are also capable of storing energy, such as starch and glycogen [4]. Polysaccharides are large molecules of high weight formed by repeating units of the same or different monomers and linked by glycosidic bonds [6]. The molecular structure of the polysaccharide varies depending on the specific source, the plant part/organism used, and the extraction method applied [11]. Polysaccharides are biocompatible, renewable, CO_2_ neutral, and fully biodegradable [12]. Taking into account these characteristics, these polysaccharide-derived polymers are used in many fields such as food packaging, tissue engineering, and bioplastics formation [13]. Most commonly, polysaccharides are derived from plants, as cellulose, hemicelluloses, lignin, pectin, alginate, and starch. 

Cellulose is one of the best-known biopolymers. It is one of the most abundant molecules on Earth since it is the main component of plant cell walls. This polysaccharide is formed by repeated units of glucose, and it can be found in natural fibers. It is not soluble in water and is not digestible by humans. However, certain animals such as ruminants and termites are able to digest it with the aid of some microorganisms [4]. Hemicelluloses, which serve as a matrix for cellulose microfibrils, are another important biopolymer and one of the main components of plant cells. About 20–30% of the dry plant biomass is made up of hemicelluloses. Pectin is also present in the primary cell wall of plants, and it acts as a structural and developmental polysaccharide. Alginate is another biopolymer obtained from seaweeds, where it is found in the cell walls and offers flexibility and strength [14]. On the other hand, starch is a semi-crystalline macromolecule composed of two polysaccharides: amylose and amylopectin [15].

In some cases, these polysaccharides are derived from several sources at the same time, such as chitin. Chitin is of animal and vegetable origin, being one of the key elements found in the cell walls of mushrooms and the epidermis of arthropods [16,17]. Chitin is biodegradable and inert in the mammalian gastrointestinal tract due to the existence of chitinases, which are extensively distributed in natura and found in bacteria, fungi, plants, and digestive systems of many animals [18]. However, chitosan, which is the main derivative from chitin deacetylation, is a biopolymer with interesting applications in food, agriculture, biomedicine, and pharmaceutical fields [19,20,21,22,23,24].

On the other hand, protein derivatives are one of the three primary types of renewable biopolymers, and they are made up of a series of amino acids that are joined together though peptide bonds. Soy protein, maize zein, wheat protein, cottonseed protein, and sunflower protein are examples of plant protein that can be utilized to make bio-based polymers. Certain animal proteins can also be used as raw materials for biological polymers, such as blood meal, gelatin, collagen, keratin, gluten, egg protein, or whey protein, among others. These residues can be used for the manufacture of protein-based biopolymers [25,26,27]. Protein-derived polymers have certain disadvantages such as inferior mechanical properties and high sensitivity to moisture compared to petroleum-derived polymers. In addition, they are difficult to process without the presence of plasticizers or pre-treatment. A particularly effective and flexible tool to improve the protein character is the modification of the functional groups of amino acids using physical (temperature or pressure) and chemical (crosslinking) methods. Other techniques have been looked into such as denaturing the original protein in its crystallized state, cleaving disulfide bonds, and grafting [1,28]. However, protein-based biopolymers offer attractive properties for future applications in the packaging industry such as biodegradability, biocompatibility, strong thermal resistance, as well as water-proof character [29]. 

Gluten is a by-product of the starch processing of wheat flour. Even though these proteins are mostly used as storage proteins in wheat plants, gluten has special viscoelastic characteristics that make it interesting in structural applications such as film forming. Specifically, gluten derived from wheat is one of the most popular choices because it is annually renewable as well as being a low-cost raw material [30]. Collagen is a potentially useful biomaterial with advantageous properties such as high availability, biodegradability, non-toxicity, biocompatibility, high tensile strength, and minimal extensibility [31]. This protein is extensively used in the food and cosmetics sectors [29]. Gelatin is a functional water-soluble protein obtained from animal by-products by acid or alkaline hydrolysis. It is mainly present in animal skins, bones, and tendons [32,33]. Its use as a gelling, thickening, or stabilizing agent is increasing, in addition to having interesting viscoelastic properties. It also has gel- and film-forming capabilities and biodegradability, and is an inexpensive and easily available material [14,34].

Biopolyesters are a group of polymers produced by several microbes and plants, being examples of materials that combine the desired benefits of plastics without harming the environment [35,36]. They contain functional groups in their chains. Through the polymerization of biopolyesters, biodegradable, renewable, and bioassimilable materials are obtained. Although it depends on their nature, most polyesters are water resistant [37,38]. Among the most recognized natural polyesters are polylactic acid (PLA), polyhydroxyalkanoates (PHAs), and polycaprolactone (PCL) [39]. PLA is a natural polyester obtained from renewable materials such as fermented sugar cane, cassava, and corn starch. In the environment, PLA-degrading microbes are abundant. Through polymerization, PLA becomes a biodegradable material. The fields where it is most widely used are the pharmaceutical and food industries [39,40]. PHAs are the only aliphatic polyesters completely synthesized by microorganisms. Over 30% of the bacteria that live in soil are capable of synthesizing PHA. Many bacteria can produce PHA in activated sludge, high seas, and other extreme environments [41]. They are often used in the biomedical field as their degradation, biocompatible, and physicochemical properties allow for drug release [39,42]. Another class of polyester is PCL, which is synthesized through the catalytic polymerization of a cyclic ester known as caprolactone. It is characterized by high permeability, slower degradation, and hydrophobic character. Unlike the rest, it is considered an implantable biomaterial since it has been maintained for long periods of time in implants. Other fields of applications are pharmacology and tissue engineering [39,43]. 

One of the main advantages of using this type of biopolymer is that they can be composted. Composting is a process in which biodegradable waste is managed in aerobic conditions. It is a fast method and requires less artificial heating, as it generates much more heat. For a polymer to be compostable, it must comply with a series of conditions set out in the ASTM D6400, ISO 17088, and EN 13432 standards: the polymer must already be biodegradable (ASTM 5338), disintegrate in a composting environment setting without posing a threat to the environment or having a negative impact on the finished compost, and as well completely degrade to CO_2_, water, and biomass with no visible remnants left behind [44,45].

## 3. Methods of Biopolymer Processing 

The biopolymer functionality depends on several factors apart from their structure and composition, such as the type, quality, and quantity of the solvent used and the processing technique used to build the final structure that will determine the interaction of the materials [11]. The main processing techniques for bio-based polymers from renewable sources are discussed in detail below.

### 3.1. Solvent Casting Method

**Figure 1:**
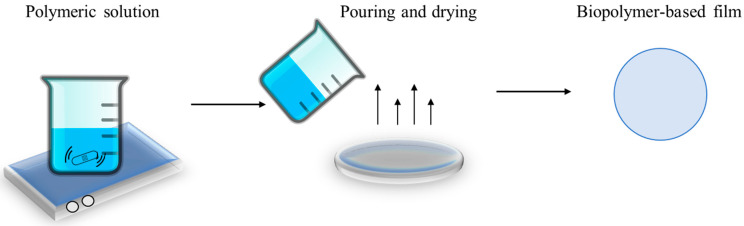
Solvent casting method scheme.

The solvent casting method, also known as the solution function or wet processing method, consists of creating an aqueous or hydroalcoholic mixture with the presence of the biopolymer. This method is based on the use of a solvent that allows the polymer to be suspended in a film-forming solution followed by solvent evaporation and polymer chain reformulation [46]. Alcohol, water, or other organic solvents are commonly used to dissolve the selected polymer. Sometimes, for the best results, the suspension polymer solution is heated, or the pH adjusted. The polymer–solvent mixture is poured into a mold, drum, or flat surface, where it is left to dry for a specific time. Once the solvent is completely evaporated, the polymeric matrix is formed and can be peeled off from the mold [47,48]. Figure 1 shows an outline of the solvent casting method. 

Casting is a simple method. However, there are a series of requirements to consider when applying the technique. One of the most important points is the solvent chosen to dissolve the polymer. If the polymer–polymer attraction forces in a solution are weaker than the polymer–solvent interactions, the chain segment will be stretched by solvent molecule diffusion. This results in swelling of the polymer matrix with the solvent. However, it should be noted that the dissolution capacity of a polymer varies depending on the solvent, hence the choice of solvent is an important point [48]. 

The molecular weight of the polymer to be used is another important point of this process. The molecular weight of the polymer affects the rate of solvent penetration. It was shown that higher molecular weight polymers dissolved more slowly compared to lower molecular weight polymers [49]. This is attributed to the fact that the high molecular weight polymer chain undergoes a slower rate of relaxation because it has greater entanglement in the chains. High molecular weight chains are unable to contract rapidly during environmental cooling, thus generating a larger free volume. For that reason, the higher the molecular weight, the greater the solvent penetration rate [50]. Moreover, this kind of biopolymer has sufficient cohesive strength and coalescence capacity. Another important requirement is that the polymer must be soluble in a volatile solvent or water. For best results, a stable solution with a suitable viscosity should be created [48]. The temperature and humidity of the environment are critical points for the process to develop correctly [51].

The main advantage of the casting method is the ease of developing the method without the need for special and expensive equipment [52]. As casting is a wet process, there is better particle–particle contact, which results in more homogeneous particle packaging and smaller and fewer defects [53]. On the other hand, a disadvantage of this method is the restrictions on the shape of the final product, which is usually simple sheets and shapes [54]. Perhaps the biggest challenge is to apply the solvent method on an industrial scale, since multiple variables, such as temperature and humidity, can cause variations in the quality of the final product [55].

Table 1 shows a compilation of the main applications of biopolymers according to the production method. For instance, the solvent casting method has been widely applied to the preparation of active or smart films for food applications. Examples include the development of carboxymethyl-cellulose/starch/purple sweet potato anthocyanin films that change color when fish begins to have pH/ammonia levels typical of contamination [56] or a chitosan-based film with the addition of sandalwood essential oil to retard lipid oxidation of butter [57]. Polymeric films based on cellulose nanocrystals and lignin nanoparticles were combined with poly(lactic acid) to provide antibacterial activity against a pathogen (*Pseudomonas syringae)* attacking tomatoes [58]. Film characteristics such as density, compactness, porosity, permeability, flexibility, and brittleness are all influenced by the polymer matrix’s degree of cohesion [46,59]. Cellulose derivatives and starch are the most commonly used polysaccharides for the manufacture of these films. Nevertheless, the low elasticity of the materials is a significant drawback that restricts their use. In order to facilitate the correct development of the process, tricks are used, such as cosolvent systems or the addition of additives such as wrecking agents, plasticizers, etc. [59]. The main non-volatile plasticizers used are glycerol, sorbitol, propylene glycol or polyethylene glycol [60,61]. However, due to its great availability, excellent plasticizing efficacy, and low exudation, glycerol is the plasticizer most commonly used [62].

Although film formation for food applications is the most common application, this technique is used in other areas. Multiple studies related to the biomedical field apply the solvent casting method to develop novel films. An alginate-based film enriched with aloe vera gel and cellulose nanocrystals as a wound dressing was developed [63]. Additionally, a biopolymer film based on keratin, fibrin, gelatin, and mupirocin that served as a substitute for wound healing was evaluated [64]. In the field of drug delivery, the solvent casting technique was used to create transdermal patches containing insulin–chitosan nanoparticles, achieving a controlled release of the insulin [65]. The casting method was also applied to biopolyesters such as PCL/PLA, where once dissolved in chloroform they gave rise to scaffolds for tissue engineering. These scaffolds showed a better biological behavior, with a reduced pore size that favors cell growth [66].

### 3.2. Coating Method

**Figure 2:**
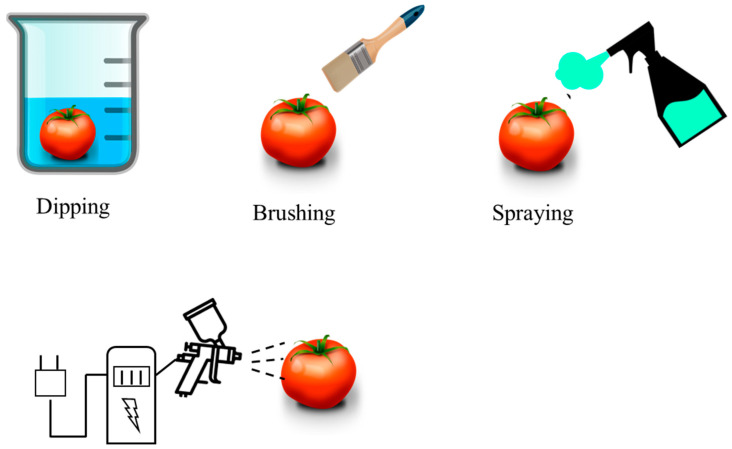
Diagram of the different coating methods.

Coating consists of the application of a solution-based coating made of a polymer or a mixture of components. This solution is applied on a surface, usually called a substrate. The coating method differs from the solvent casting method in the way of application. The purpose of the coating can be technical, decorative, or both [102,103]. Within the coating method, varieties can be found: the dipping process is the oldest technique and consists of immersing the product in the coating solution. It is commonly used in the food industry, for example, for coating vegetables and fruits to act as barrier against moisture and gases [104,105]. The brushing process is when the coating is applied directly to the surface of the product with a brush or brushing equipment [106,107]. In the spraying process, the final product is sprayed with many drops of the coating solution. Hydraulic spray nozzles, high-pressure spray guns, or air atomization systems are often used to facilitate the process [108]. In electrical spraying, the coating solution passes through the atomizer nozzle connected to a source of high electrical potential [109]. Figure 2 shows the coating method and its different modalities. 

Generally, the coating method has three important stages [110]:

- immersion or permanence time: the substrate is plunged into the precursor solution at a constant speed to give the substrate enough interaction time with the coating solution (in the dipping method). After this, it is left to stand for a certain time.
- Deposition and drainage: a thin layer of the precursor solution is entrained by drawing the substrate upward at a constant rate. Water that is in excess will drain off the surface.
- Evaporation: the solvent evaporates, creating a thin film. This process can be accelerated by hot drying.

Coatings can be made from biological materials such as lipids, polysaccharides, and proteins. Polysaccharides and proteins are interesting materials since they can form cohesive molecular networks through strong interactions between molecules, such as hydrogen bond, van de Waals interactions, or crystallization. These molecular cohesions give the coating a good barrier property to gases (mainly O_2_ and CO_2_), and good mechanical properties [111,112,113]. Polyols that act as plasticizers (e.g., glycerol or polyethylene glycol) or acid/base substances (e.g., acetic or lactic acid) that control pH are typically included as minor components. The biopolymers applied in the coating method are usually prepared in aqueous solutions. However, there is a lack of studies on the influence of the solvent applied in the coating process.

The solubility of the biopolymers together with the viscosity of the final solution depends on the molecular weight of the polymer. A high polymer molecular weight results in a more viscous solution, but also decreases the solubility of the polymer [114]. On the other hand, creating a coating that adheres properly to the surface of the final product requires an estimation of the interfacial tension between the coating solution and the product surface [115]. For compatibility between the surface and the solution, an interesting action is to reduce the surface tension of the coating solution. This reduces the interfacial tension and improves adhesion between the product and the coating [116].

A positive aspect of the coating method is that this is a process where it is not necessary to spend large amounts of substrate since the layers that are created rarely exceed micrometers of thickness. It is also a simple and inexpensive method compared to other polymer production methods, in addition to having a high adhesion capacity in the coating of complex geometries [117]. Although it is a method that brings great benefits, it also suffers from certain disadvantages that diminish its reliability. For instance, there are negative thermal effects such as cracking or delamination, effects due to lack of atmospheric protection (e.g., penetration of contaminants into the substrate), and limitations of the coating materials, such as different melting points, availability in various forms such as sheets/powders, biocompatibility between the components of the coating solution, among others [117].

There are multiple studies where the biopolymer coating technique is applied. The main field of application is the food industry. In this field, edible coatings are applied directly to food and consumed as part of it. They have been used to prevent moisture migration or create shiny surfaces to make food more appealing to consumers. Most of the studies conducted on foods are on fruits and vegetables. For instance, an edible alginate-based coating for application on fresh-cut watermelon was used [67]. The coating maintained quality and sensory acceptability, as well as extended the shelf life of the food product. Chitosan-based coatings have also been developed to delay the ripening of fruits such as guava and prolong their quality [68]. Gelatin-based coatings incorporated with *Mentha pulegium* essential oil significantly inhibited the presence of molds and yeasts in strawberries stored at 4 °C [69]. 

On the other hand, materials engineering based on polymer coating for drug loading/release, cell immobilizations/protection, or antibacterial/antiviral applications is an area of great interest. For instance, a cellulose nanofibril-based coating was applied on a 3D scaffold obtained from bone marrow, thus promoting adhesion, proliferation, and osteogenesis of bone tissue cells [70]. Likewise, TiO_2_-SiO_2_/chitosan–lysine-based coatings were loaded with a drug to combat osteomyelitis. The study showed that the drug was released correctly and could be used as a drug delivery vehicle [71]. PCL dissolved in dichloromethane was used as a coating to improve the strength of a bone implant screw. The study in rats concluded that thicker, denser bone had formed around the PCL-coated screw in the animals’ femurs [72].

### 3.3. Electrospinning Method

**Figure 3:**
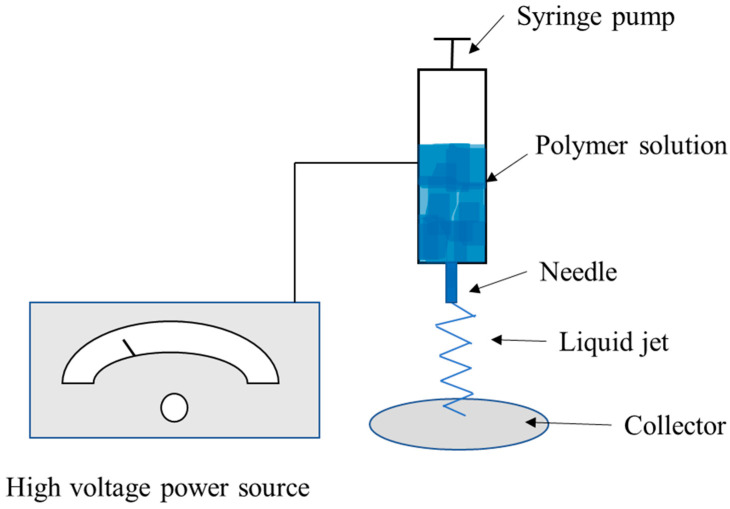
Diagram of the electrospinning method.

Electrospinning is a technique in which a high electrical voltage created between two electrodes is used to generate charged strands of polymer at a specific flow rate. These fibers typically range in diameter from 2 nm to several micrometers [118]. The polymer solution is drawn through a nozzle with a high-voltage electric field using mechanical pressure combined or by gravity alone. This is followed by solvent evaporation, which causes the production of solid fiber [119,120]. Figure 3 shows a schematic of the electrospinning process. Generally, ordinary basic electrospinning equipment consists of four main elements:

- an electric field, which is applied on the needle containing the solution with which the spinning is performed, and the collector connected through two electrodes.
- A flow-controlling syringe pump for supplying the rotating solution.
- A syringe with a capillary tube or spinneret to hold the spinning fluid and a metal needle with a flat end.
- An electrically conductive collector (target) present in various shapes.

Although it is a relatively simple method, there are several parameters that influence the subsequent characteristics of the fibers generated. These parameters to consider could be classified into three areas: solution, process, and environment. The cleanliness and safety of electrospinning are closely related to the solvent used in the solution, which can be water or organic solvents. The application of water as a solvent is becoming more and more frequent. However, this greatly restricts the choice of polymeric materials due to the low presence of water-soluble polymers [121]. In addition, the final product should undergo crosslinking to provide stability to the water present in the nanofibers [121]. Low-solubility solvents are also recommended, since solutions with better electrospinning capacity are obtained [122]. In addition, the surface tension of the solvents is important. By lessening the surface tension of the solution, bead-free fibers can be produced. For instance, cellulose fibers using two different solvents, acetone and dimethylacetamide, were manufactured [123]. The authors observed that when the solvents were used individually the fibers had beads. However, when applied together, the fibers were produced without beads. The viscosity of the solution is another key point that affects the subsequent behavior of the process and the quality of the product obtained. The viscosity of the solution is related to the molecular weight of the solute and the concentration of the polymer used [124]. Low molecular weight polymer solutions form beads instead of fibers, therefore it is interesting to use high molecular weight solutions with a suitable viscosity to create the fibers correctly [118].

Regarding the electrospinning process, the voltage applied directly affects the fiber diameter. If the applied voltage is high, a large volume of polymer solution is expelled, resulting in larger diameter fibers [125]. Additionally, the flow rate through the syringe nozzle will influence the final morphology of the fibers. If the flow velocity is high, the solvent evaporation rate is lower, and this means inadequate drying with the presence of beads on the fibers. On the other hand, if the speed is low, the solvent will have more time to evaporate. It is important to find the right speed point to obtain the best result [118,126]. 

Temperature and humidity are among the parameters to be considered in relation to the environment while fabricating electrospun fibers. Studies showed that at higher temperature the viscosity of the solution decreases, generating fibers of smaller diameter. On the other hand, the higher the humidity in the environment, the smaller the pores in the fiber structure [127,128]. In addition, humidity influences the evaporation of the solvent as well. A relatively high humidity is recommended because if the humidity is too low, the solvent can cause clogging at the needle tip of the equipment [118,129,130].

The polymeric fibers generated from this process have a large specific surface area, high porosity, and high absorbency capacity, which makes them interesting for applications that it will be mentioned below [127]. Electrospinning can be used with both synthetic and natural polymers, however, the latter have received a lot of attention in recent years [131,132]. Natural polymer nanofibers have properties such as low antigenicity or antimicrobial capacity, that make them interesting for many applications. However, the main limitations of natural polymers are that they are more difficult to process and have poorer mechanical properties. Therefore, synthetic and natural polymers are usually used together to improve the properties of the fibers.

Compared to other methods, electrospinning is easy to handle, cost effective, with a high loading capacity, and applicable at room temperature. Additionally, the micro/nanofibers produced by the electrospinning method have shown distinctive characteristics, such as a high surface/volume ratio, significant porosity, adjustable mechanical characteristics, and changeable morphologies [124,133]. Although successful fabrication and manufacturing of electrospun nanofibers have been carried out continuously, some critical drawbacks are associated with the processing parameters such as the viscosity of the solution and surface tension, among others [134].

Originally, this technique was used in the textile industry [135], but nowadays it is an interesting method for the economical and efficient manufacture of food packaging. According to the literature, polysaccharide and protein-derived polymers are the most widely used polymers in the electrospinning method. Specifically, collagen, gelatin, chitosan, cellulose, and alginate are the most used. Biomedical applications and wound healing are the most common fields where this technique is applied. For example, electrospinning has developed collagen-based fibers that showed improved healing progression [136]. The high porosity of biopolymer nanofibers favors gas exchange through the wound, preventing desiccation and dehydration [137]. Chitosan-coated collagen nanofibers enabling bone regeneration in a mesenchymal stem cell study were developed [76]. Additionally, nanofibers created from polycaprolactone and natural silk fibroins were shown to have high cellular biocompatibility and to promote cell growth of damaged bone tissue [77]. Gelatin/polyvinyl alcohol/chondroitin sulfate nanofibrous scaffolds for skin tissue engineering were also developed [74]. PCL was used as a material in the electrospinning technique for the creation of scaffolds that favored the growth and proliferation of corneal keratocytes. This use is interesting for corneal wound healing [78]. More studies are needed on the use of polymeric fibers made by electrospinning in the food industry. For instance, zein/sodium alginate nanofibers were developed [75] that were shown to exhibit antioxidant/antimicrobial effects and could be applied as an active inner layer of laminated films for food applications.

### 3.4. Three-Dimensional Printing Method

**Figure 4:**
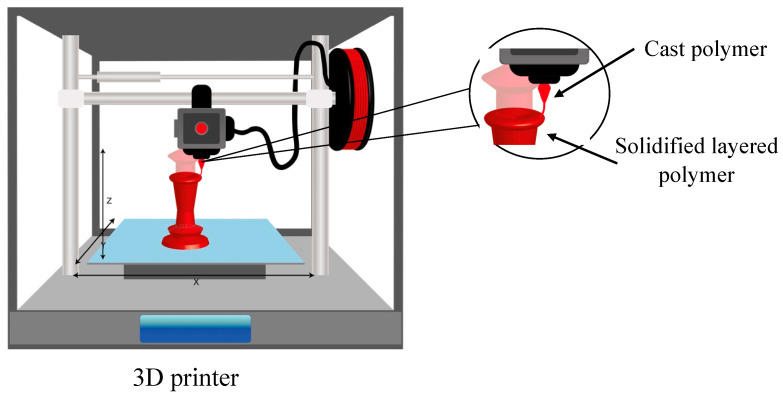
Schematic diagram of the 3D printing method.

Three-dimensional printing makes it possible to manufacture objects by layering a material with the help of a print head and a nozzle. The selected material is applied on a substrate with a specific geometry that has been pre-designed [138]. Then, during the construction process, the different layers of the selected material are poured in. Finally, the structures are removed from the support. Depending on the type of printing, a curing phase may be necessary [139]. Figure 4 shows the process carried out by the 3D printer. 

Several essential requirements must be met for the equipment to be properly used in 3D printing procedures. The pressure-driven extrusion flow rate depends on the viscosity of the biopolymer used and, if it reaches the required volume flow rate under conventional system pressure, it is a suitable material. The formation of a bead with a table geometry is another key point, which depends on the superficial energy of the system. However, it is normal for certain residual stresses to appear within a component that is formed layer by layer [140]. 

Assuming these requirements are met, it is important to know the physicochemical characteristics of the biopolymer to be used as printing ink. First of all, the thermal property is the ability of the material to melt or solidify and, consequently, the transition temperature of the biopolymer from the fluid to the solid state must be known [141]. Rheological properties refer to the ability of the biopolymer to flow. For instance, it is sometimes of interest that they exhibit plastic-like behavior, with the fluid flowing through the nozzle but solidifying rapidly when the nozzle pressure is removed [142]. Likewise, the ability of the material to flow through the nozzle when a force is applied, also known as slip or surface property, is a point to be considered. The lower the surface tension of the material, the more likely it is to spread when it comes into contact with a surface [143]. 

Currently, the adoption of this technique remains complex due to the scarcity of printable materials that are environmentally friendly and meet performance and manufacturing requirements [144]. Moreover, biopolymers cannot be used in their natural form, and it is an effort to convert them into raw materials for this technique. Certain biopolymers have inherently poor physicochemical properties, such as insolubility in common solvents, which complicates the process [145]. However, 3D technology has interesting advantages, such as the fabrication of complex structures and the fabrication of customized designs composed of different components [146]. In addition, material waste is reduced, making manufacturing more cost effective [138,145,147]. Among the most important limitations are the time required to perform the technique and the cost of the equipment [148]. 

This technique has important applications that can revolutionize sectors such as medical applications. Complex constructions can be made using 3D printing technology to replace or repair worn-out bone and cartilage tissues. For the repair of these, biopolymers such as alginate are frequently utilized [79,149,150]. A collagen–alginate combination was tested as bioink for the creation of cartilage [79]. This cartilage had favorable mechanical strength and biological functionality [79]. Another biopolymer commonly used in this technique is chitosan. A chitosan hydrogel scaffold through 3D printing was developed [80]. A biopolymer-based scaffold with remarkable adhesion and proliferation capacity of human fibroblasts was achieved. Additionally, starch is made up of heat- and pressure-sensitive molecules, which facilitates the depolymerization process produced in 3D printing. This depolymerization modifies the structure and the physical and chemical properties of the biopolymer, making it interesting for applications such as functional food [144]. As a biopolyester, PLA was the material used for the bioprinting of tubular scaffolds for application in bone tissue engineering. The application of this material showed stable mechanical and thermal properties over time [83]. Three-dimensionally printed starch beads for the release of bioactive compounds in foodstuffs were manufactured [81]. The porosity feature could be attractive for application in bioactive compound/drug delivery in the food or pharmaceutical industry. In addition, this biopolymer has potential applications in the food industry. Starch is frequently used as a thickening/gelling agent or a rheological modifier in the field of 3D food printing. Sweet potato-derived starch as a structural enhancer of three-dimensional printing was used [82]. Its application even extends to the manufacture of customized foods, where packaging based on cellulose was developed for use with foods with low moisture content [151].

### 3.5. Injection Molding Method

**Figure 5:**
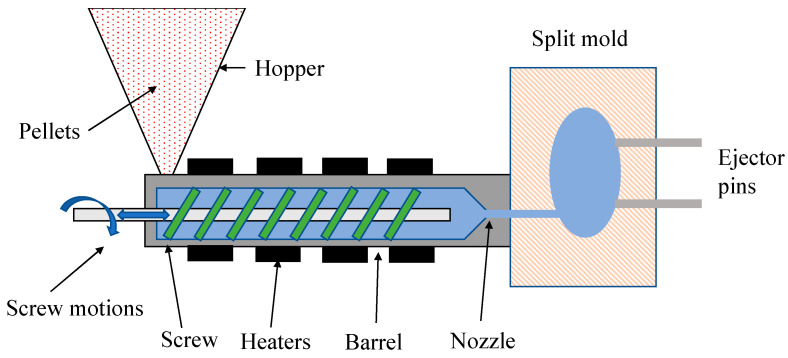
Schematic diagram of the injection molding method.

Injection molding is one of the most widely used methods for manufacturing biopolymer-based products. Figure 5 shows the injection molding process. This process consists of three steps [152]:

1. filling: the polymer is melted at high temperature and quickly fills a cold mold to form a cavity with the desired shape of the product.
2. Packing/holding: the pressure is increased, and additional material is pressed into the mold to offset the effects of temperature decline and crystallinity growth on density during the solidification.
3. Cooling: this stage begins when solidification occurs at the entrance of the cavity. From this point on, no more material will enter or leave the mold, and the retaining pressure can be released. The time to eject the mold is when the solid layer of the surface reaches a sufficient thickness to provide rigidity.

Like the other methods, this process causes important changes in the properties related to the rheology and thermomechanics of the biopolymers due to stress variations during the process, high temperature, and cooling rate of the final product [153]. Therefore, it is important to thoroughly analyze the factors that affect injection molding before deciding to manufacture a product. These factors can be divided into two categories [154]:

- machine parameters: these include the barrel, nozzle, and coolant temperatures. Pressure is influenced by packaging pressure, back pressure, and injection pressure. It is also important to take into account the movement, switching point, injection speed, and shot volume.
- Process parameters: these include the mold temperature and the melting and cooling temperature in addition to injection, holding, and cooling time, mold opening speed, injection, and heat/cooling dissipation.

Part dimensions, sink marks, resistance to weld lines, and other aesthetic defects such as irregular textures or marks are parameters used to determine part quality indexes [154]. 

The application of biopolymers in this technique is limited since the melting temperature used in this method is many times higher than the decomposition temperature of biopolymers such as chitosan. However, a chitosan/thermoplastic starch-based polymeric matrix was developed and subjected to injection molding followed by molding compression [84]. Starch is another biopolymer characterized by low mechanical properties and high water absorption. Sodium alginate in cassava starch was applied to injection molding, creating a composite that showed good compatibility between the two phases and improved mechanical properties [85]. On the other hand, the PLA biopolyester has favorable mechanical properties compared to other biopolymers, making it interesting to use it in this technique. Stereocomplex PLA formulations subjected to injection molding at different mold temperatures were produced. PCL combined with crayfish meal was used to create biocomposites. An improvement in the mechanical properties of the systems with the presence of PCL was observed [87]. This process is also carried out for the development of PLA/PHA nanocomposites. Although PHA has a similar chemical composition and flow temperature to PLA, it is not processable. However, when combined with PLA, it acts as a nucleating agent, which means that it improves the mechanical properties and the barrier behavior of the material [88]. 

### 3.6. Compression Molding Method

**Figure 6:**
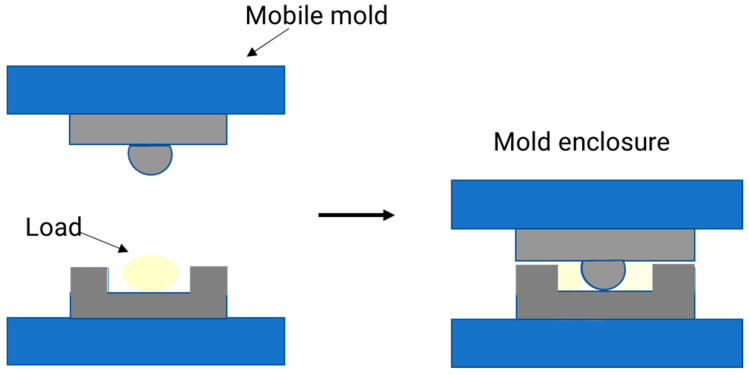
Scheme of the compression molding process.

The compression molding technique, also known as press molding, consists of placing the desired biopolymer (in the form of granules or sheets) between two molds heated to high temperatures. In this way, the material acquires its final shape after pressure is exerted on the molds and then the final matrix is cooled and removed after it has cured [9,155,156]. Figure 6 shows the compression molding process.

This technique is a secondary process because the materials used in this technique must undergo pre-processing [157]. This process consists of four main steps [155]:

- pre-charge and placement preparation: a certain amount of biopolymer is placed in the pre-heated mold. At this point, the biopolymer is called pre-load or load, and it must be weighed before being placed in the mold. The dimensions of the charge are set to cover 50% of the mold surface. The position of the pre-load in the mold is also a key point. If it is improperly placed, it will affect the quality of the piece.
- Mold enclosure: when the pre-charge is put in the mold, the upper mold swiftly descends to touch the top surface of the pre-charge. Usually, the mold descends at a speed of 5–10 mm/s to compress the load. The performance of the process and the quality of the final product are significantly influenced by the mold temperature and closure speed.
- Curing: after the pre-charge material has been fully inserted into the mold cavity, the mold is kept closed as the molding pressure is maintained for a certain period. With this, the biopolymer cures and the final product is obtained. The time it takes to cure depends on the biopolymer itself, the thickness of the piece to be created, and the temperature at which the mold is heated.
- Part release: when the piece has solidified, it is removed from the mold. After this step, the part is left at room temperature to cool.

Some crucial steps must be considered during the development of this method. Prior to molding, the removal of moisture from the polymer through heat is an important step. The moisture content of the material influences the final structure and affects the content and size of the pores. According to the studies, 80 °C is the ideal temperature for the process of removing moisture from natural fibers. A vacuum furnace is one of the pieces of equipment suggested for this step [158,159,160]. On the other hand, the duration of this process in natural fibers is also a point to consider. According to several articles, 24 h was established as the recommended duration of this process [161,162]. 

Besides that, control parameters in the compression molding technique to develop superior and desired composite properties are molding temperature, compression pressure, and heating and compression duration. First, the appropriate molding temperature is between the melting temperature of the polymer matrix and the degradation temperature of the natural fiber. A lower temperature will facilitate wetting of the fibers and a higher temperature may affect the polymer properties. Studies have shown that the degradation temperature of most natural fibers is approximately 200 °C [163,164]. On the other hand, high compression pressure is crucial to improve the interfacial bond between the matrix and the fiber [165,166]. According to the literature, the pressure used in composite materials from natural fibers varied from 2 to 69 MPa [167,168,169,170,171]. The use of one pressure or another depends on the material, hence it must be controlled since this parameter directly affects the mechanical properties of the final material [159]. Finally, heating and compression duration of the process is also an important factor. For best results, most studies set the temperature and pressure holding time for natural fibers at 4 to 15 min [167,168,169,172]. In general, the final step related to curing is usually performed at 150–180 °C. However, the pressure and time to be applied vary depending on the type of biopolymer used and the desired thickness [48].

The compression molding process has some advantages such as the high reproducibility of the pieces generated from this method, the creation of complex shapes, or the minimal loss of material. It also has a relatively high production rate as the mold cycle time requires only a few minutes, hence it can be applied at an industrial scale. However, the price of compression molds is more expensive compared to other equipment applied in other techniques [91,155,159].

According to the revised bibliography, natural polymers derived from proteins and polysaccharides are the most widely used. These are polymers that have intermolecular bonds that undergo multiple interactions, which broaden their functional properties [173]. This technique is applied mainly in the manufacture of films. Chitosan films using citric acid as a crosslinker were developed [89]. In addition to obtaining films with excellent mechanical properties, applying this technique as a production method reduced production time and avoided the use of organic solvent by mixing the polymer and acid in its solid form. The same technique was followed to created edible films based on wheat gluten protein [90]. In addition, this technique was applied to create active films based on cassava starch and chitosan [91]. These films were able to extend the shelf life of pork slices stored under refrigeration. On the other hand, fish gelatin previously subjected to extrusion and then compression molding was applied to create films [92].

### 3.7. Extrusion Method

**Figure 7:**
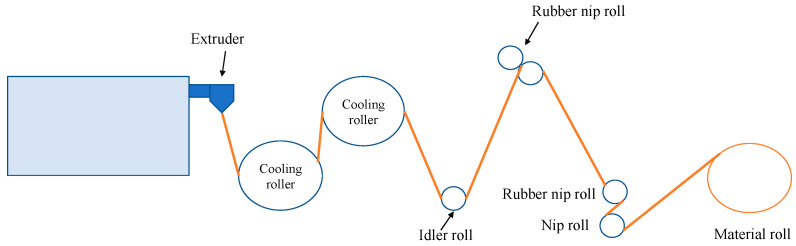
Scheme of the extrusion process.

The extrusion process is a method in which a material is forced to flow under various conditions through an orifice of a certain shape and at a pre-determined rate to obtain a product. Figure 7 shows the extrusion process. During the process, thermal and shear energies are applied to the material. This generates transformations of the structural, chemical, and nutritional profile, such as gelatinization and starch degradation, protein denaturation, and lipid oxidation [174]. Extrusion is a method commonly used in the polymer industry. The food and pharmaceutical industries are other fields of application, where this technique is used to modify the microstructure or chemistry of polymers. For example, the most commonly extruded food material is starch. In this case, the starch granule is broken to make it digestible. It is also common for flavors, nutrients, and pharmaceuticals to be encapsulated by this method [175]

The parameters to be taken into account in this method are grouped into three categories depending on [176]:

- process: related to raw material characteristics, moisture content, barrel temperature, screw speed and configuration, and die dimension.
- System: including the specific mechanical energy used, temperature, viscosity, pressure, and residence time distribution.
- Product: such as color, nutrition, texture, flavor, and fiber formation.

With these extrusion parameters, the properties of the final product will be affected.

Within this technique is a variation known as cast film extrusion. This is one of the processes used to produce polymeric films, especially for the packaging or coating industries. The process consists of a film extruded through a hanger die, stretched in air between the die and a cooling roller, or between the die and a water bath. Normally, the stretching distance is a few centimetres [177]. As mentioned above, starch is one of the most commonly used materials in this technique. Starch/gelatin films were developed. Glycerol and sorbitol were added as plasticizers, as they are known to interact strongly with starch at the molecular scale, forming hydrogen bonds and improving its structure [93]. Chitosan/thermoplastic corn starch was used as a blend to produce films. The analysis showed homogeneous films with no presence of granules from starch and chitosan. In addition, the films improved their extensibility and thermal stability despite decreasing their mechanical properties [97]. Additionally, biopolyesters are used. For instance, PLA extrusion is the preferred method for high-throughput production for packaging applications [94]. PLA films with α-tocopherol, butylated hydroxytoluene, and poly-ethylene glycol were developed using a cast film extruder. The presence of PLA in the film composition caused water vapor permeability and decreased oxygen permeability, in addition to excellent antioxidant properties [95]. The combination of starch with biopolyesters has also been studied. PHA-based films were developed with the addition of thermoplastic starch as a plasticizing agent. The final film exhibited excellent mechanical properties for flexible packaging in addition to reducing aging, which is an inherent problem of PHA/starch-based materials [96]. 

### 3.8. Graft Copolymerization Method 

**Figure 8:**
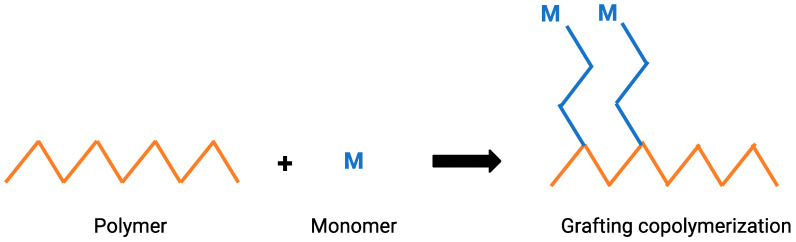
Graft copolymerization process.

The most efficient technique to combine the properties of two or more different kinds of polymers in a single unit is by graft copolymerization [178]. Figure 8 visually shows the chemical process to which the polymers are subjected in this process. Graft copolymerization is currently the subject of intense investigation as a useful technique for changing the properties of natural biopolymers to create newer polymeric materials with hybrid qualities [179]. Therefore, graft copolymerization in natural polymers has been studied to modify the structures of these polymers. There are two main types of grafting: “grafting to”, a method where end-functionalized polymer molecules react with complementary functional groups located on the surface to form tethered chains, while “grafting from” makes use of the polymerization that attaches initiating groups on the substrate surface [180]. 

Like the other techniques discussed, this method also has several factors to be taken into account for the process to develop properly. The nature of the backbone to which the monomer is to be bonded (physical nature, chemical composition, etc.) plays an important role in the process. For instance, cellulose is resistant to grafting reactions in the presence of water due to its insolubility [181]. However, the presence of a suitable solvent can enhance the mobility of the radicals to the active sites on the substrate backbone to achieve engraftment [180]. The reactivity of the monomer is also an important point. The polar and steric character, swellability of the backbone in the presence of the monomers, concentration of monomers, and others all affect how reactive monomers are. It has been observed that grafting efficiency increases with higher monomer concentration, but this occurs up to a certain point, then begins to decrease. This behavior reflects an initial increase in monomer concentration in the vicinity of the backbone, accelerating the homopolymerization reaction in place of the graft [182,183].

On the other hand, the solvent is the carrier with which the monomers are carried to the vicinity of the backbone. The main effect of the solvent is to loosen the polymeric network to allow the grafting reaction to take place [184]. The solubility of the monomer in the solvent, the miscibility of the solvents if more than one is employed, and the formation of free radicals are some of the factors that affect the solvent choice. Water has a negligibly low side reaction involving chain transfer due to the zero-chain transfer constant of water. Therefore, it is considered an interesting solvent to use in this method. Apart from the solvent used, all grafting reactions require an initiator with which the percentage of grafting is determined [185]. According to studies, it has been observed that once a certain concentration of the initiator is obtained, further increasing its concentration does not increase the conversion of the grafted monomer [186,187]. Additionally, the solubility of this initiator is important, ideally it should be fully soluble, and it can initiate the grafting reaction through the monomers. Additives such as metal ions, acids, or organic salts are often added to improve the grafting performance or degree of copolymerization [182]. Finally, the temperature at which the method is performed is another key point, as it controls the kinetics of graft copolymerization. Generally, the higher the temperature, the higher the graft yield. This may be because the diffusion processes of the monomers in the backbone are faster at a higher temperature [183].

Chemical modification of polymers by grafting is another way to improve its performance and broaden its potential applications. Grafting copolymerization is applied in many fields such as chemical engineering, dyeing, biomaterials, drug delivery, foods, agriculture, papermaking, and wastewater treatment [178]. In most of the studies performed on the grafting method, chitosan was the most studied biopolymer. Chitosan has two reactive groups that can be grafted: on the one hand, free amino groups on deacetylated units, and on the other hand, hydroxyl groups on carbons. Chitosan grafting allows the formation of functional derivatives through the covalent attachment of a molecule to the chitosan backbone. Recent research shows that with the application of this method, chitosan obtains a much improved water solubility capacity and bioactivities such as antibacterial and antioxidant activities [188,189]. A chitosan graft was synthesized, showing that it could become a potential material for biomedical applications [98]. Its application in water treatment was studied through grafting which introduced an optical indicator for cadmium ions into a chitosan/cellulose acetate blend-based membrane [99]. 

Lignin is another polymer that has been extensively studied. Lignin has hydroxyl groups in its composition, which are highly reactive and easy to modify. With the grafting method, lignin is functionalized with active sites to obtain versatile properties [190]. The grafting of lignin side chains through phenol oxidases to obtain a lignin-based bioadhesive to be applied in the wood industry was studied [100]. On the other hand, cellulose is a polymer characterized by its sensitivity to water/moisture absorption. This poor chemical resistance inhibits its use in outdoor applications and, consequently, surface modification is important [191]. Graft copolymerization of cellulose is usually performed with hydrophobic monomers that incorporate the desired functions, improving physical–chemical and mechanical properties. A study conducted on cellulose fibers grafted with poly(butyl acrylate) showed an increase in thermal, acid, and base resistance compared to raw fibers in addition to decreasing water and moisture absorption, thus proposing a promising application as a green composite [192,193].

As with the other techniques, there are certain drawbacks in using this method. For example, the removal of unreacted side chains is necessary and should be taken into account before finalizing the process. Nevertheless, this method allows preparation of complex graft copolymers and thus broadens the applications of graft copolymers in a variety of fields [194].

## 4. Conclusions and Future Perspectives

In recent decades, the interest in biopolymers has increased. Natural polymers have demonstrated abundant availability, compostability, low cost, and ecological characteristics that make them superior to synthetic materials. The drivers behind the growth of compostable biopolymers vary across countries, frequently referring to restrictions on the use of conventional plastics, bio-based products, and zero-waste programs Nature offers many biopolymers with differences in properties and molecular structure. These properties can be controlled through the choice of processing technique and the parameters that affect it. The performance of these biopolymers depends on several factors, such as chemical composition, physical properties, biopolymer modification techniques, the additives applied, structural defects, and environmental conditions of the compound.

Current technological improvements have helped biopolymer end products scale up to broader purposes and they could soon have their performance level on par with petroleum-based synthetic polymers. However, studies on the use of biopolymers in processing techniques and the development of new methods are required.

## Figures and Tables

**Table 1:** Methods of biopolymer processing and applications. CMC is carboxymethylcellulose, PLA is polylactic acid, PHA is polyhydroxyalkanoate, and PCL is polycaprolactone.

Production Method	Biopolymer	Application	References
Solvent casting	CMC, starch	Detection of content of total volatile basic nitrogen value in contaminated fish	[56]
	Chitosan	Retard lipid oxidation of butter	[57]
	Alginate	Wound dressing application	[63]
	Keratin, gelatin	Wound healing recipe for in vivo studies	[64]
	Chitosan PCL/PLA	Controlled release patches for insulin Engineering of scaffold for tissue engineering	[65] [66]
Coating	Alginate	Extend shelf life of fresh-cut watermelon	[67]
	Chitosan	Delays ripening and reactive oxygen species in guava fruits after harvesting	[68]
	Gelatin	Bioactive packaging to prolong the shelf life of strawberries	[69]
	Cellulose	Bone tissue regeneration in vivo	[70]
	Chitosan PCL	Antiosteomyelitis drug release and bone repair Bone healing and osteogenesis promoter	[71] [72]
Electrospinning	Chitosan	Release of paclitaxel to kill prostate cancer cells	[73]
	Gelatin	Scaffold for skin tissue engineering	[74]
	Alginate	Antioxidant/antimicrobial active packaging	[75]
	Chitosan, collagen	Guided bone regeneration	[76]
	Silk fibroin PCL	Promoting bone cell growth Scaffolds promoting corneal keratocyte growth and proliferation	[77] [78]
3D printing	Collagen, alginate	Bioink for the creation of cartilaginous tissue	[79]
	Chitosan	Cell adhesion and growth, tissue engineering	[80]
	Starch	Beads for bioactive compound release	[81]
	Starch PLA	Structural enhancement for 3D printing of surimi Tubular scaffolds for bone tissue engineering	[82] [83]
Injection molding	Chitosan Starch PLA PCL PLA/PHA	Development of matrix biopolymer for agricultural/packaging applications Creation of cassava starch/sodium alginate composites Development of high-temperature-resistant stereocomplex PLA Creation of crayfish protein–PCL biocomposite material Development of PLA/PHA nanocomposites	[84]
			[85]
			[86]
			[87]
			[88]
Compression molding	Chitosan	Film production	[89]
	Wheat gluten	Film production	[90]
	Cassava starch	Film production for cold-stored pork meat slices	[91]
	Fish gelatin	Film production	[92]
Extrusion	Starch/Gelatin PLA PLA PHA Starch/Chitosan	Film production Film production Development of antioxidant film Development of PHA/thermoplastic starch film Development of corn starch/chitosan film	[93] [94] [95] [96] [97]
Grafting copolymerization	Chitosan	Biomedical field	[98]
	Chitosan	Water treatment for the removal of heavy metals	[99]
	Lignin	Wood composite manufacturing	[100]
	Cellulose	Green composite applications	[101]

## Data Availability

Data sharing not applicable.
